# Effective *in vivo *and *ex vivo *gene transfer to intestinal mucosa by VSV-G-pseudotyped lentiviral vectors

**DOI:** 10.1186/1471-230X-10-44

**Published:** 2010-05-11

**Authors:** Hiroshi Matsumoto, Takahiro Kimura, Kazunori Haga, Noriyuki Kasahara, Peter Anton, Ian McGowan

**Affiliations:** 1 Department of Medicine, Division of Digestive Diseases, University of California Los Angeles (UCLA) David Geffen School of Medicine, Los Angeles, CA, USA; 2 Department of Molecular & Medical Pharmacology, University of California Los Angeles (UCLA) David Geffen School of Medicine, Los Angeles, CA, USA; 3 Magee-Womens Research Institute, Division of Gastroenterology, Hepatology, and Nutrition, University of Pittsburgh School of Medicine, Pittsburgh, PA, USA

## Abstract

**Background:**

Gene transfer to the gastrointestinal (GI) mucosa is a therapeutic strategy which could prove particularly advantageous for treatment of various hereditary and acquired intestinal disorders, including inflammatory bowel disease (IBD), GI infections, and cancer.

**Methods:**

We evaluated vesicular stomatitis virus glycoprotein envelope (VSV-G)-pseudotyped lentiviral vectors (LV) for efficacy of gene transfer to both murine rectosigmoid colon *in vivo *and human colon explants *ex vivo*. LV encoding beta-galactosidase (LV-β-Gal) or firefly-luciferase (LV-fLuc) reporter genes were administered by intrarectal instillation in mice, or applied topically for *ex vivo *transduction of human colorectal explant tissues from normal individuals. Macroscopic and histological evaluations were performed to assess any tissue damage or inflammation. Transduction efficiency and systemic biodistribution were evaluated by real-time quantitative PCR. LV-fLuc expression was evaluated by *ex vivo *bioluminescence imaging. LV-β-Gal expression and identity of transduced cell types were examined by histochemical and immunofluorescence staining.

**Results:**

Imaging studies showed positive fLuc signals in murine distal colon; β-Gal-positive cells were found in both murine and human intestinal tissue. In the murine model, β-Gal-positive epithelial and lamina propria cells were found to express cytokeratin, CD45, and CD4. LV-transduced β-Gal-positive cells were also seen in human colorectal explants, consisting mainly of CD45, CD4, and CD11c-positive cells confined to the LP.

**Conclusions:**

We have demonstrated the feasibility of LV-mediated gene transfer into colonic mucosa. We also identified differential patterns of mucosal gene transfer dependent on whether murine or human tissue was used. Within the limitations of the study, the LV did not appear to induce mucosal damage and were not distributed beyond the distal colon.

## Background

Gene transfer to the gastrointestinal (GI) mucosa is a therapeutic strategy which could prove particularly advantageous for treatment of various hereditary and acquired intestinal disorders, including inflammatory bowel disease (IBD), GI infections, and cancer [1-5].

Non-viral vectors for delivery of exogenous DNA are limited by low efficiency of transduction *in vivo*, and do not provide long-term expression [6]. First-generation retroviral vectors can achieve long-term expression through their ability to integrate permanently in the genome of target cells, but gene transfer to the GI tract by these vectors was also found to be inefficient [7-10]. Conversely, adenoviral vectors can infect a wide range of cells, including intestinal epithelial cells, and show high levels of transgene expression [11,12], but these are non-integrating vectors and so expression is transient [13]. Furthermore, adenoviral vectors induce robust cellular and humoral immune responses *in vivo*, resulting in elimination of transduced cells and neutralization of the vector upon repeat administration [14]. In contrast, adeno-associated virus (AAV) vectors lack all viral genes and have limited capacity to induce cell mediated immune responses. In addition, AAV may have the potential to transduce intestinal crypt progenitor cells resulting in extended transgene expression [15].

LV, such as those derived from human immunodeficiency virus (HIV), are distinct from earlier generation retroviral vectors in their ability to infect quiescent cells through active import of the viral preintegration complex across the intact nuclear membrane, even in post-mitotic cells [16,17]. LV pseudotyped with the vesicular stomatitis virus envelope glycoprotein (VSV-G) exhibit an expanded host range that allows entry into most cell types in a wide variety of species ranging from zebrafish to man [18]. Highly efficient gene delivery by VSV-G-pseudotyped LV has been reported in various types of terminally differentiated primary cells *in vitro *and *in vivo*, including neurons, hepatocytes, cardiomyocytes, vascular endothelium, alveolar pneumocytes, and keratinocytes [19-29]. With regard to intestinal cells, these vectors have been shown to be capable of transducing human and canine colonic epithelial cell lines via the apical membrane in polarized monolayer cultures *in vitro *[30]. However, to date there have been no studies evaluating VSV-G-pseudotyped LV for gene delivery to primary intestinal cells, especially those subjacent to the mucosal epithelia, particularly in the context of the architecturally complex native tissue.

Therefore, in these studies we tested the ability of VSV-G-pseudotyped LV to deliver reporter genes to colonic mucosa via the apical surface *in vivo*, after intraluminal instillation per rectum in a murine model, and *ex vivo* in a human intestinal explant system [31].

## Methods

### Vector construction

**Figure 1:**
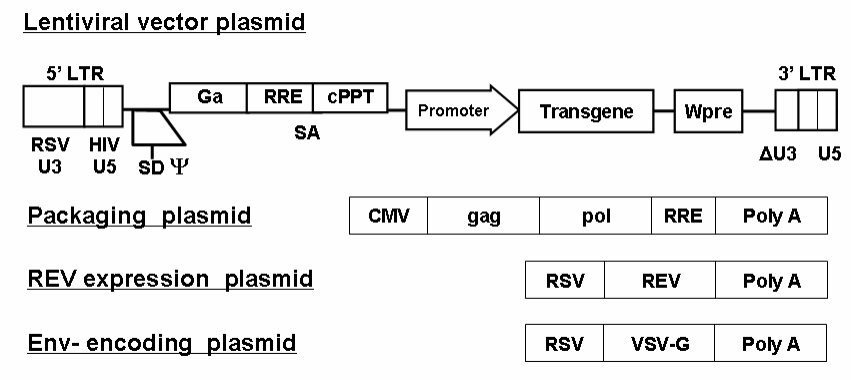
**Lentiviral vector (LV) constructs and *ex vivo *explant culture system**. (A) Schematic representation of self-inactivating vectors containing a central polypurine tract (cPPT)/central termination sequence, immediate early cytomegalovirus (CMV) promoter. Vectors were constructed for expression of beta-galactosidase (β-Gal) and firefly luciferase (fLuc).

The pRRLsin-hCMV-β-Gal vector was constructed by insertion of the β-Gal reporter gene from plasmid pSV-β-Gal (Promega, Madison, WI, USA), and the pRRLsin-hCMV-fLuc vector was constructed by insertion of the fLuc gene [32], respectively, into the multiple cloning site (MCS) of pRRLsin-hCMV-MCS-pre, a third-generation, self-inactivating LV construct kindly provided by Dr. Luigi Naldini (University of Milan, Italy) [33] (Figure 1A).

### Cell culture, virus production, and *in vitro *gene transfer

Human embryonic kidney cell line 293T, and human colon cancer cell lines Caco-2, LoVo, and WiDr, (ATCC, Manassas, VA, USA), were cultured in Dulbecco's modified Eagle's medium (DMEM), Ham's F12K, or RPMI-1640 medium, respectively, with 10% fetal bovine serum and 1% penicillin-streptomycin. LV virus was produced in 293T cells using a third-generation packaging system as previously described [34], using either pRRLsin-hCMV-β-Gal or pRRLsin-hCMV-fLuc, to produce LV-β-Gal or LV-fLuc, respectively. Virus titers were determined by HIV-1 p24 ELISA (Perkin Elmer, Waltham, MA, USA) and expressed as p24 equivalent units (ng/ml). Vector transductions were performed on 1 × 10^5 ^target cells with 8 μg/mL polybrene (Sigma, St. Louis, MO, USA) at 37°C. Polybrene is a small, positively charged molecule that binds to cell surfaces and neutralizes surface charge and has been shown to enhance cell transduction by retroviruses [35].

After replacement of the medium and further incubation for 24 hr, cells were washed twice in phosphate-buffered saline (PBS), fixed in 2% formaldehyde/0.2% glutaraldehyde (Sigma, St. Louis, MO) for 10 min at room temperature, and stained with 20 mg/ml X-Gal solution (5-bromo-4-chloro-3-indolyl beta-D-galactoside; Sigma, St. Louis, MO, USA) at 37°C for 2 hr.

### *In vivo *studies

All *in vivo *studies were performed according to institutional guidelines under protocols approved by the UCLA Animal Research Committee. Briefly, 6 to 8-week-old female BALB/c mice (Charles River Laboratories Inc., Wilmington, MA, USA) were divided into a non-treated control (NC) group, and two groups that received intrarectal instillation of either LV or vehicle solution (DMEM). Prior to instillation, anesthetized mice were given an intrarectal enema of 50% ethanol (vol/vol in distilled H_2_O). Pretreatment with ethanol enemas has been shown to increase intestinal transduction with other vectors [36]. Two hours after the enema, 100 μL of vehicle or viral vector solution containing a total of approximately 1000 ng p24 equivalent units was instilled intrarectally. The mice were inverted for 30 sec after administration of intrarectal products to prevent leakage.

### Macroscopic assessment and *ex vivo *bioluminescence imaging

Health status and body weight was monitored throughout the study. On days 2, 7, and 21 after vector or vehicle instillation, cohorts of mice were sacrificed, and the entire colon was removed. Colon length from cecum to anus, weight, and a macroscopic colonic damage score were recorded The macroscopic colonic damage score was based on the degree of tissue adhesion, mucosal ulceration, and intestinal wall thickness (Table 1) [37].

**Table 1:** Macroscopic Colonic Damage Score System

Tissue Adhesion	
*Score*	*Observation*
0	No adhesion
1	Little effort required to separate the colon from the surrounding tissue
2	Moderate effort required to separate the colon from the surrounding tissue
3	Severe adhesions
**Degree of ulceration**	
***Score***	***Observation***
0	Normal appearance of the colon
1	Focal hyperemia with no ulcer
2	Presence of an ulcer and inflammation
3	Two (2) or more ulcers and regions of inflammation

**Wall thickness**	
***Score***	***Observation***
0	Normal thickness
1	Mild thickening
2	Moderate thickening
3	Severe bowel thickening

*Ex vivo *bioluminescence imaging was performed 2 days after rectal instillation of LV-fLuc using the Xenogen IVIS system (Caliper Life Sciences, Alameda, CA, USA). Bioluminescent signal intensity was expressed as photons per second per cm^2 ^per steridian (p/s/cm^2^/sr), and color images were processed with Living Image and IGOR-PRO analysis software (Wave Metrics, Portland, OR, USA).

### Histological evaluation and X-Gal histochemical staining

For routine histology, colon and other tissues (spleen, liver, lung, kidney) were fixed in 4% paraformaldehyde overnight, placed in 30% sucrose/PBS for 2 hr, and embedded in OCT compound. Serial 5-μm cryosections were stained with hematoxylin/eosin (H&E) for evaluation of histopathology score. Mucosal inflammation was scored using a semi quantitative technique (Table 2) [38].

**Table 2:** Histological Scoring System

Infiltration of inflammatory cells	
*Score*	*Observation*
0	Rare inflammatory cells in the lamina propria
1	Increased numbers of inflammatory cells in the lamina propria
2	Confluence of inflammatory cells extending into the submucosa
3	Transmucosal infiltrates
**Tissue Damage**	
***Score***	***Observation***
0	No mucosal damage
1	A discrete lymphoepithelial lesion
2	Surface mucosal erosion
3	Extensive mucosal damage and ulceration

For X-Gal histochemistry, tissues were fixed in 2% glutaraldehyde for 2 hr, embedded in OCT compound, and 10-μm cryosections were stained in 1 mg/ml X-Gal solution at 37°C for 24sr, rehydrated, and counterstained with 0.1% Nuclear Fast Red (Sigma, St. Louis, MO, USA). The number of positive staining cells was counted in five independent fields in random areas on two non-consecutive slides at 200× magnification.

### Real-time quantitative PCR (qPCR) analysis

Quantification of vector copy number was performed at each time point by TaqMan qPCR assay to detect the HIV-1 packaging signal sequence, using 300 ng genomic DNA (equivalent to 5 × 10^4 ^genomes) isolated from murine colon and other tissues, including stomach, small intestine, liver, kidney, spleen, lung, heart, brain, and bone marrow [39]. A reference curve was prepared by amplifying serial dilutions of LV-encoding plasmid in a background of genomic DNA isolated from untransduced murine colon. Genomic DNA from a PC3 cell line previously confirmed by flow cytometry to be 100% transduced by LV-GFP vector was used as a positive assay control.

### Human colorectal tissue explant culture and *ex vivo *gene transfer

All human endoscopic biopsies were collected from healthy HIV-negative volunteers at UCLA Medical Center, Los Angeles, USA. The protocol for the use of human endoscopic biopsies was approved by Institutional Review Board of the David Geffen School of Medicine at UCLA (#02-05-001-13).

Explant cultures were established as previously described [31]. Briefly, explants were placed on presoaked Gelfoam^® ^(Pharmacia and Upjohn Company, Kalamazoo, MI, USA) rafts with the epithelium uppermost. Tissues were transduced by pipetting LV solution and polybrene (8 μg/mL) onto the top of each Gelfoam^®^-supported explant. After 2 hr incubation, tissues were washed three times, placed on fresh Gelfoam^® ^rafts, and incubated at 37°C for a total of 24 hr. Explants were then either fixed in glutaraldehyde, embedded in OCT, and 10-μm cryosections prepared for X-Gal as above, or were fixed in formaldehyde, embedded in OCT, and 7-μm sections prepared for immunofluorescence staining.

### Immunofluorescence staining and quantitative morphometry

The identity of transgene-expressing cells in harvested murine colon tissues or human colorectal explant tissues was examined by immunofluorescence double-staining with antibodies against β-Gal and cell-specific phenotypic markers. Briefly, serial 7-μm cryosections were stained for E. coli β-Gal (Promega, Madison, WI, USA). Additional staining included antibodies directed against human and murine cytokeratin AE1/AE3 and CD45 (Dako North America Inc., Carpinteria, CA, USA); and CD4, CD8, and CD11c (BD Pharmingen, Franklin Lakes, NJ, USA). β-Gal staining was then visualized with Alexa Fluor^® ^594. Cytokeratin and CD45 were visualized with Alexa Fluor^® ^488 (Invitrogen Corporation, Carlsbad, CA, USA). The CD4, CD8, and CD11c antibodies were already conjugated with Alexa Fluor 488. The Vector mouse-on-mouse (M.O.M™) Immunodetection Kit (Vector Laboratories, Burlingame, CA, USA) was used to avoid the high background staining caused by antibody binding to endogenous murine immunoglobulin when secondary antibodies were used to amplify primary murine antibodies. Slides were mounted and nuclei counterstained using VECTASHIELD^® ^and DAPI (4',6-diamidino-2-phenylindole; Vector Laboratories, Burlingame, CA, USA). In order to quantify LV-mediated β-Gal transduction efficiency, the total number of cells showing co-localization of β-Gal-positive and CD45 or CD4 positive signals in 3 randomly selected fields per section at 200 × magnification were identified, and expressed as percentages of the total number of β-Gal-positive cells or the total number of CD45 +/CD4+ cells.

### Statistical analysis

All values were expressed as means ± SD. Comparisons between groups were made using the Student *t*-test and the Mann-Whitney U test, and *p *values <0.05 were considered statistically significant.

## Results

### LV-mediated gene transfer to human colorectal cancer cell lines *in vitro*

**Figure 2:**
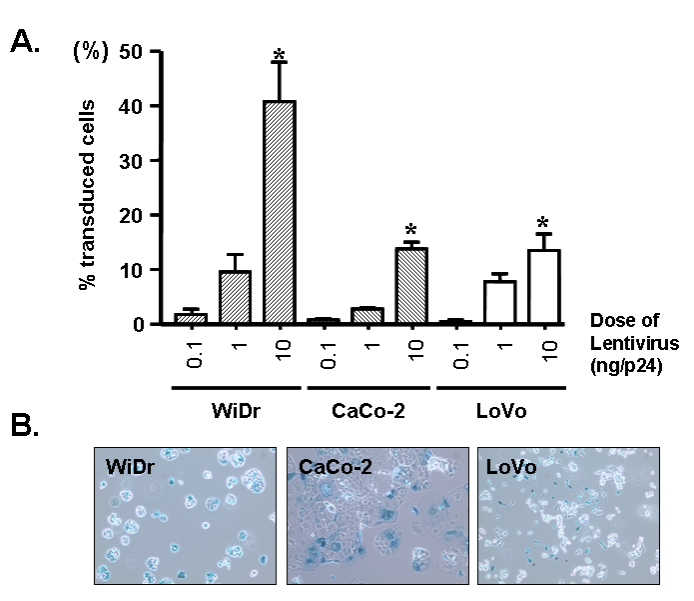
***In vitro *transduction of vesicular stomatitis virus G protein (VSV-G)-pseudotyped lentivirus (LV) encoding β-Gal in colonic cell lines**. (A) Percentage of cells transduced by the VSV-G-pseudotyped LV. One hundred cells were counted in three randomly selected, non-adjacent fields in triplicate. (B) Colon adenocarcinoma cell lines showing evidence of β-Gal transduction following exposure to 10 ng p24 of vector. All values were expressed as means ± SD. *p < 0.05 compared with the results of 0.1 ng p24 VSV-G LV transduction.

LV-mediated transduction efficiency was first assessed *in vitro *using human colorectal adenocarcinoma cell lines Caco-2, WiDr, and LoVo. In each cell line, a dose-dependent increase in the number of cells showing positive signals upon X-Gal staining was observed with increasing LV concentration (Figure 2A, B). The highest dose tested was 10 ng p24 equivalent units of LV, which corresponds to a biological infectious titer of 10^**6 **^transducing units (TU) on a standardized cell line such as 293T [40]. At this dose, transduction rates per 1 × 10^5 ^target cells were 40.6% for WiDr, 13.7% for Caco-2, and 13.3% for LoVo, indicating that although these colon adenocarcinoma cell lines are less permissive for LV compared to 293T embryonic kidney cells, significant gene transfer can be achieved with higher multiplicities of infection. Seppen et al. have previously reported higher rates of transduction in Caco-2 cells but used a transwell system with GFP expressing first and third generation lentiviral vectors [41].

### Administration of LV for transduction of murine colorectal mucosa *in vivo*

The safety of gene transfer procedures using LV expressing either fLuc or β-Gal was assessed by both macroscopic and histopathologic criteria in three experimental groups; mice receiving the LV, mice receiving a mock installation (the viral plasmid solution in DMEM), and a control group (N = 8 per condition) Intrarectal administration of 1000 ng p24 equivalent units of VSV-G-pseudotyped LV, corresponding to a 293T cell-based standardized biological titer of 10^**8 **^TU, did not affect body weight or induce any gross abnormalities upon routine observation. The ratio of colon weight-to-length was 0.33 ± 0.04 in the non-treated control (NC) group, 0.34 ± 0.5 in the mock instillation control group, and 0.27 ± 0.4 in the LV instillation group. Thus, there were no significant differences among these three groups in macroscopic damage assessment or histopathological evaluation scores (Additional File 1). More specifically, the use of ethanol enemas did not appear to induce significant mucosal damage. No pathological findings could be observed in kidney, spleen, lung, and liver harvested from control and LV-treated mice at each time point (data not shown).

### *Ex vivo *bioluminescence murine imaging following rectal instillation of LV-fLuc

**Figure 3:**
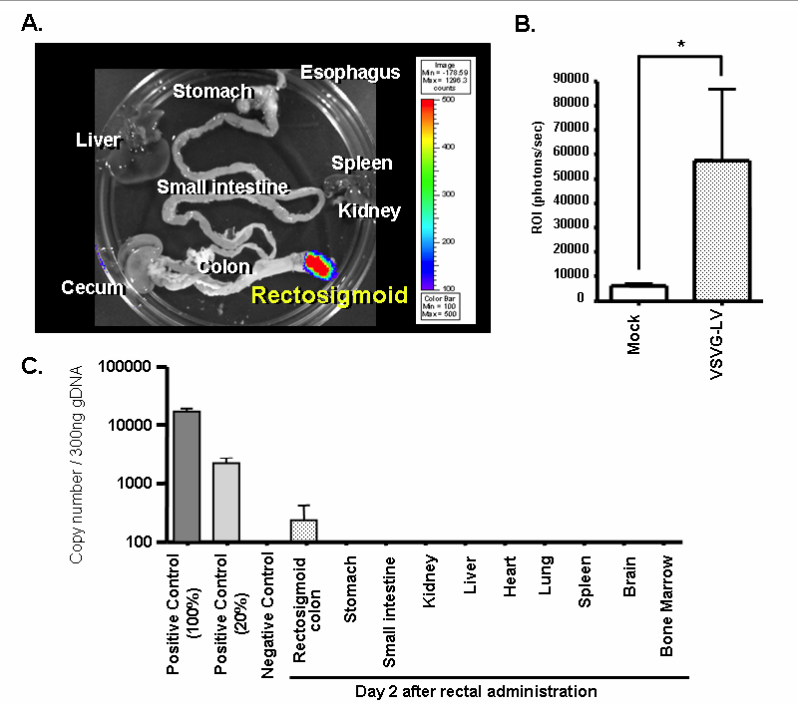
**Bioluminescence imaging analysis**. (A) *Ex vivo *bioluminescence imaging analysis after rectal administration of vesicular stomatitis virus-G protein (VSV-G)-pseudotyped lentivirus (LV) expressing firefly-luciferase (fLuc). This pseudocolor image, which is superimposed on a grayscale reference image, uses color (blue, least intense; red, most intense) to illustrate signal strength. (B) Photon emission (p/s/cm2/sr) in the region of interest (ROI) of colon transduced by VSVG-pseudotyped LV shows significantly higher levels of transduction compared to the mock control. (C) Biodistribution of VSV-G LV after rectal administration demonstrated by real-time quantitative polymerase chain reaction (qPCR) analysis of viral copy number in 300 ng of genomic DNA of *in vivo *(equivalent to approximately 50,000 cells). The detection limit was 50 copies per 300 ng genomic DNA.

Strong positive signals were observed in the distal colon adjacent to the rectum in 8/8 (100%) of mice exposed to VSV-G-pseudotyped LV encoding the fLuc reporter gene (Figure 3A). Quantitative measurement of bioluminescent photon emission from LV-transduced distal colon showed a significantly higher level of transduction than that of mock-treated colon (57560 ± 28960 p/s/cm^2^/sr for the LV instillation group vs. 6260 ± 813.3 p/s/cm^2^/sr for the mock instillation group; p < 0.001) (Figure 3B). Additional studies are needed to determine whether the physical distribution of the ethanol enemas and/or the LV influenced the location of the bioluminescence signal.

### Real-time qPCR analysis of LV transduction in murine colon tissue

Real-time qPCR was employed to quantify the vector copy number of LV integrated into genomic DNA from murine colon tissues after *in vivo *administration. Using spiked samples to obtain a reference curve, this method was determined to be sensitive enough to detect 50 copies of LV per 5 × 10^4 ^cellular genomes. Genomic DNA from both positive control cells (data not shown) and transduced rectosigmoid colon showed amplification of proviral LV sequences. The average copy number per 300 ng genomic DNA from LV-transduced colon was 231.3 ± 183.7 (Figure 3C). As expected, no detectable qPCR signals were observed in genomic DNA from colon tissues of non-treated or mock-treated controls. Importantly, even in LV-treated animals at 2 days post-vector instillation, no detectable qPCR signals were found in any extra-intestinal tissues examined, including stomach, small intestine, liver, kidney, spleen, lung, heart, brain, and bone marrow (Figure 3C). As the LV was not treated with DNAse prior to injection, we cannot exclude the possibility that the data in Fig 3C in part reflect plasmid contamination.

### X-Gal histochemistry and double immunofluorescence staining of LV-βGal transduction in murine colon tissues

Histochemistry was performed using optimized X-Gal concentrations and pH conditions to minimize background staining due to endogenous mammalian β-galactosidase in the GI tract. As expected, under these conditions, no staining was observed in mouse colon tissues from non-treated and mock-treated control groups, nor in the non-intestinal tissues (kidney, spleen, lung, and liver) from any animals including the LV-treated group.

**Figure 4:**
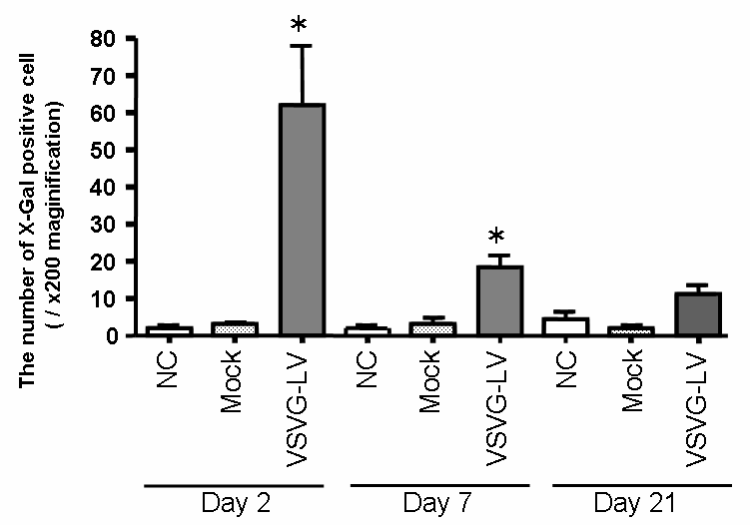
**X-Gal histology in *murine *colon transduced by vesicular stomatitis virus G protein (VSV-G)-pseudotyped lentivirus (LV) encoding β-Gal *in vivo***. The time course of β-Gal expression in the colon of BALB/c mice. Each group had 6 mice. The colon was removed at the indicated time points. The number of X-Gal-positive cells in murine colon transduced by LV-β-Gal *in vivo*. All values were expressed as means ± SD. *p < 0.05 compared with the results of normal control (NC).

**Figure 5:**
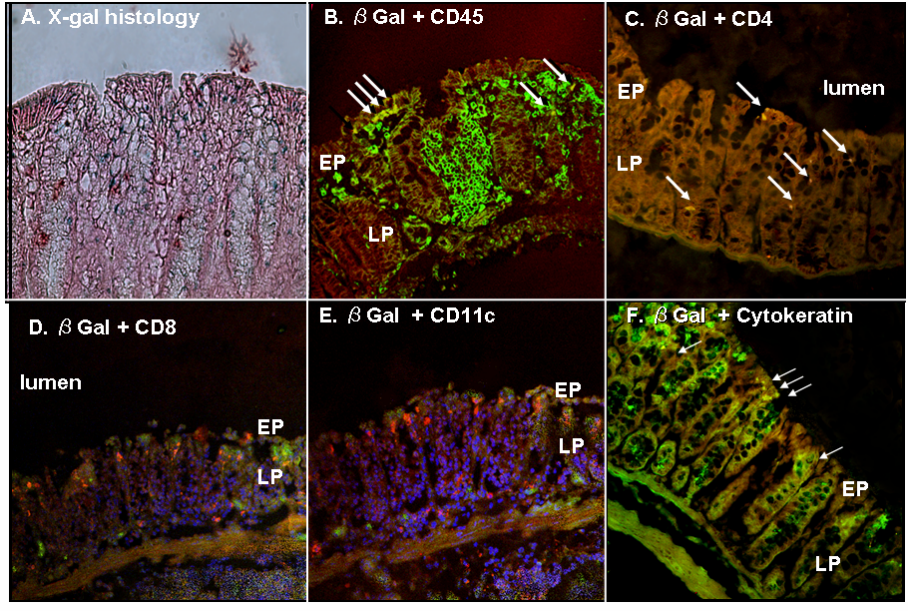
**Double immunofluorescence histology analysis in *murine *colon**. (A) Histochemical analysis of transduced murine colon *in vivo*. The X-Gal-positive cells were evident mainly on the luminal surface of the distal colon (magnification × 400). (B-F) Double immunofluorescence staining of tranduced murine colon examined under confocal microscopy. On Day 2 following rectal administration, β-Gal-positive cells in murine colon tissue expressed CD45, CD4, and cytokeratin, but not CD8 or CD11c. In these photomicrographs, β-Gal-positive cells are visualized by red fluorescence, CD phenotypes and cytokeratin are visualized by green fluorescence, and all images display fluorescence overlays: (B) β-Gal and CD45; (C) β-Gal and CD4; (D) β-Gal and CD8; (E) β-Gal and CD11c; and, (F) β-Gal and cytokeratin. Double-positive cells indicated by the arrowheads are yellow. (EP; epithelial cell, LP; lamina propria cell)

In contrast, positive-staining cells were observed in colon tissues from animals treated with LV expressing the E. coli β-Gal reporter gene. The initial number of positive-staining cells in the LV-treated group was 62.3 ± 18.5 per microscopic field at 200× magnification on Day 2 post-vector instillation (Figure 4). However, the number of positive-staining cells was then observed to decrease, to 19.1 ± 5.3 per 200 × field on Day 7, and 15.7 ± 5.1 per 200 × field on Day 21. These positive-staining cells were found predominantly toward the luminal surface of the colon, but were identified in both mucosal epithelium and, importantly, the lamina propria (LP) (Figure 5A).

To further characterize the identity of the transduced cells, immunofluorescence double-staining was performed using an E. coli β-Gal-specific antibody in combination with various cellular immunophenotypic antibodies. Quantification of double-positive staining in colon tissues harvested on Day 2 post-LV instillation demonstrated that 27 ± 5% of the β-Gal-positive cells were also positive for cytokeratin. Consistent with their histological origin, β-Gal/cytokeratin double-positive cells were observed solely in the epithelial layers of colon tissues from transduced animals. Cells exhibiting double-positive staining for both β-Gal and the common leukocyte antigen CD45 were found in both mucosal epithelium and lamina propria (LP) regions. These β-Gal/CD45 double-positive cells represented 70 ± 11% of the total population of β-Gal-positive cells.

A subset of LV-transduced white blood cells was further identified to consist of T lymphocytes, dendritic cells, or macrophages, as 27 ± 2% of the total population of β-Gal-positive cells were also positive for CD4. In fact, of the total population of CD4-positive cells, 48 ± 13% were β-Gal-positive, indicating a significant transduction of half of this cell population. These β-Gal/CD4 double-positive cells were observed only within the LP of transduced murine colon. Notably, there were no cells showing co-expression of both β-Gal and CD8 or CD11c (Figure 5B-F).

### LV-β Gal-mediated *ex vivo *gene transfer to human colorectal explant tissues

**Figure 6:**
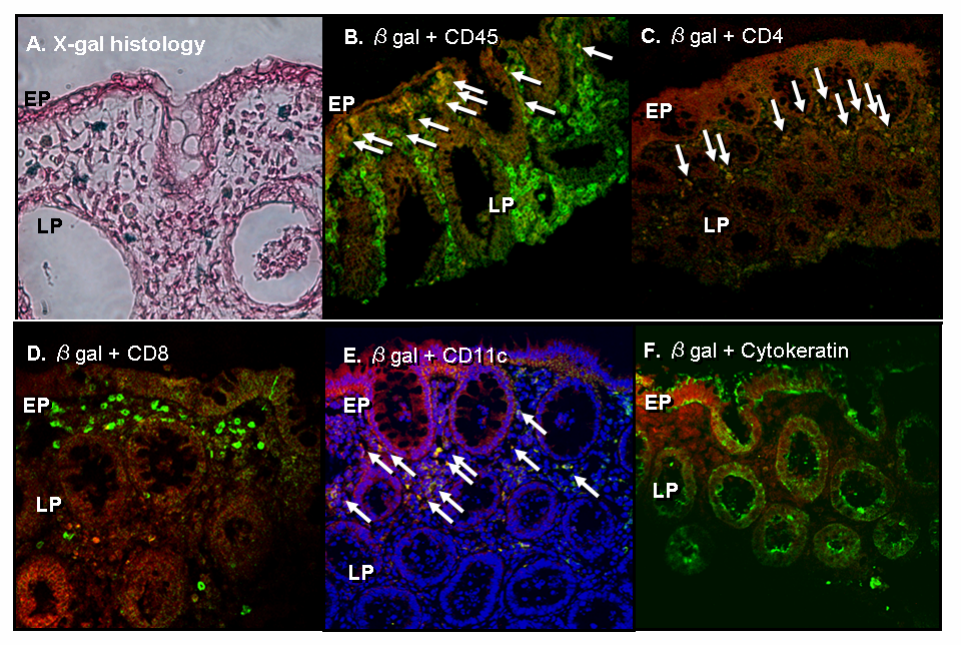
**X-Gal histology and double immunofluorescence histology analysis in *human *colonic explants transduced by VSV-G-pseudotyped lentivirus (LV) encoding β-Gal**. (A) Histological appearance of transduced human explants after X-Gal staining. X-Gal-positive cells, here stained blue, were found mainly in the LP. (B-F) Double immunofluorescence confocal microscopy of human explants transduced with VSV-G. β-Gal-positive cells expressed CD4, CD45, and CD11c, but not CD8, or cytokeratin. In these photomicrographs, β-Gal is visualized by red fluorescence, each cellular phenotype marker is visualized by green fluorescence, and nuclei are visualized by blue DAPI staining, respectively, and all images display fluorescence overlays: (B) β-Gal and CD45; (C) β-Gal and CD4; (D) β-Gal and CD8; (E) β-Gal and CD11c; and, (F) β-Gal and cytokeratin. Double-positive cells, indicated by the arrowheads, are yellow (EP; epithelial cell, LP; lamina propria cell).

Human colon tissue specimens were incubated with 1000 ng p24 units of LV-β-Gal, and examined within 24 hr of explant culture. The number of X-Gal-positive cells after transduction was 12.4 ± 1.5 per low power (×200) field. In contrast to the murine studies, the transduced cells in human samples were primarily found within the LP (Figure 6A). These data might reflect the availability of both apical and basal aspects of the explant to LV transduction, and/or the use of polybrene. By double immunofluorescence staining using human cellular immunophenotypic antibodies, 84 ± 16% of **β**-Gal-positive cells were also found to be positive for CD45. Of these, 41 ± 12% of **β**-Gal-positive cells were found to be CD4-positive, and 35 ± 8% were CD11c-positive. Notably, no **β**-Gal-positive cells expressed CD8 or cytokeratin (Figure 6B-F). Non-specific background staining was not observed using this technique (Additional File 2). This identifies transduced cells as mucosal lamina propria CD4+ lymphocytes, macrophages, dendritic cells, and other leukocytes. Consistent with the results obtained after *in vivo *LV instillation in mice, 54 ± 15% of the total population of CD4-positive cells in *ex vivo *LV-transduced human explant tissues were also positive for **β**-Gal. We did not perform immunohistochemical examination of mesenteric lymph node tissue and so we do not know whether LV transduced cells could be identified at this site.

## Discussion

Using a combination of molecular, imaging, and histological techniques, we have demonstrated for the first time that VSV-G-pseudotyped LV can mediate detectable gene transfer to architecturally intact human and murine intestinal mucosa after topical application. The overall transduction level was observed to decrease over time, with a 3-fold reduction between Day 2 and Day 7, but was then relatively consistent between Day 7 and Day 21. This may reflect both initial transgene expression from unintegrated episomal reverse-transcribed circular forms of LV provirus, which is transient in duration, as well as reduced numbers of transduced non-adherent cells (such as lymphocytes) as they migrate or circulate out of colon tissue *in vivo*, or reflect cells lost through apoptosis or karyolysis in the *ex vivo *explant model.

An additional finding is the demonstration that in human intestinal tissue, the transduced cells are predominantly in the lamina propria, an observation differing from previous beliefs that it is predominantly epithelial lineage cells that are transduced. Co-localization of phenotypic cell surface markers and transgene expression showed notable differences in the types of cells transduced between experiments involving LV-mediated transduction of murine vs. human colon tissue. Significant transduction of cytokeratin-positive cells was observed after *in vivo *transduction of murine colon, but not after *ex vivo *transduction of human explants colon tissue. Conversely, after *ex vivo *transduction of human explant colon tissue, but not after *in vivo *transduction of murine colon, we observed significant transduction of cells positive for CD11c, a cellular marker of macrophages and dendritic cells, but which is also weakly expressed on B cells, NK cells, and T cell subsets.

A number of parameters may have contributed to these differences. For example, to avoid degradation of the tissue architecture, LV-transduced human colon tissues had to be analyzed after no more than 24 hours of explant culture, and the time course for accumulating a detectable level transgene product may require a longer time interval for epithelial cells. Of course, LV transduction after rectal instillation *in vivo *also necessitates penetration from the intestinal lumen, and the mucosal epithelium represents the only tissue surface directly in contact with the virus solution. In contrast, virus applied *ex vivo *to the surface of endoscopically acquired human tissue explant samples has access to sub-epithelial cell layers that would not normally be available to virus administered intraluminally to intact intestine *in vivo*. Another difference between the *in vivo *and *ex vivo *experiments was the use of ethanol in the former, and polybrene in the latter, to facilitate lentiviral transduction. We did not explore the extent to which these different experimental parameters might influence the results of our experiments.

We did observe a difference between the present study and previous studies using explant tissues, in terms of the specific localization of transduced cells within the LP. In the present study, LV-transduced cells in human explant samples were found primarily in the subepithelial region of the LP whereas in other colorectal explant studies using adenoviral vectors [42] transduction was commonly observed in the basolateral region. However, it is difficult to compare our data with other published studies as the cell tropism may differ between vectors. In addition, differences in the histological distribution of vector-specific cellular receptors, such as the coxsackievirus/adenovirus receptor (CAR) required for efficient binding of adenovirus to target cells may influence experimental results.

There were also similarities in cell transduction results from the murine rectal *in vivo *transduction and human explant *ex vivo *transduction models. In both cases, VSV-G-pseudotyped LV exhibited the ability to transduce CD4-positive cell population. Notably, LV pseudotyped with the VSV-G envelope, which only requires binding to phospholipid constituents of most mammalian cells [43], achieved much more efficient infection of T cells than is possible with identical vectors pseudotyped with amphotropic retrovirus envelope [44]. However, CD4 is not only expressed by helper T lymphocytes and T regulatory cells, but also by macrophages and dendritic cells at lower levels. All of these cell types generally reside in the LP, which is where the predominant staining was observed in humans. It is not clear why the LV was unable to transduce CD8 positive cells and this finding warrants additional *in vitro* studies with purified T cell populations.

As the vectors used are replication-defective and can only mediate a single cycle of infection, this finding suggests that LV may be efficiently transported intact across the mucosal epithelium as has been suggested as a natural route of HIV infection. It has also been reported that dendritic cells may form projections into the intestinal lumen to sample incoming antigens, which may permit HIV infection as well as LV transduction of this cell population.

## Conclusions

In summary, these studies have demonstrated the feasibility of using VSV-G-pseudotyped LV to safely achieve appreciable levels of localized gene transfer into architecturally intact primary murine and human intestinal tissues.

## Competing interests

The authors declare that they have no competing interests.

## Authors' contributions

HM conducted the majority of the experiments described in this paper. TK carried out the molecular genetic studies, made the LV, and performed the statistical analysis of the experimental data. KH helped to analyze the *in vivo *animal data. NK provided technical assistance for the LV experiments, PA collected the endoscopic biopsies, and IM supervised the research, and edited the final manuscript^. ^All authors read and approved the final manuscript.

## Pre-publication history

The pre-publication history for this paper can be accessed here:

http://www.biomedcentral.com/1471-230X/10/44/prepub

## Supplementary Material

Additional file 1 The effect of vesicular stomatitis virus G protein (VSV-G)-pseudotyped lentivirus (LV) rectal gene transduction on healthy murine cells. BALB/c mice were divided into three groups; a normal healthy control (NC) group and two groups that received either placebo or VSV-G LV following a preliminary ethanol enema (EtOH). Mice received 1000 ng p24 VSV-G LV by rectal administration under anesthesia. (A) VSV-G LV did not affect weight loss in healthy mice. Results are shown as a percentage of original weight for each group. (B) There was no significant difference in the ratio of colon length to weight between groups. Further, there was no significant difference in (C) macroscopic damage score or (D) histomorphologic score among all groups. Click here for file

Additional file 2 The negative control pictures of immunofluorence staining using AF488 secondary antibody. Non-specific green staining is not observed in either (A) AE1/AE3 or (B) CD45 examination of murine intestinal tissue or (C) AE1/AE3 (D) CD45 examination of *ex vivo *human explant tissue Click here for file

## References

[B1] Prieto J Herraiz M Sangro B Qian C Mazzolini G Melero I The promise of gene therapy in gastrointestinal and liver diseases Gut 2003 52 Suppl 2 ii49 ii54 12651882 10.1136/gut.52.suppl_2.ii49 PMC1867750

[B2] Raper SE Wilson JM Making space for intestinal gene therapy Gastroenterology 1997 112 1753 1756 10.1016/S0016-5085(97)70060-X 9136857

[B3] Forbes SJ Hodgson HJ Review article: gene therapy in gastroenterology and hepatology Aliment Pharmacol Ther 1997 11 823 836 10.1046/j.1365-2036.1997.00226.x 9354189

[B4] van MC Te Velde AA venter SJ van De Rodriguez Pena MS Gene therapy in the treatment of intestinal inflammation Int J Colorectal Dis 2004 19 79 86 10.1007/s00384-003-0501-4 12827411

[B5] Wirtz S Neurath MF Gene transfer approaches for the treatment of inflammatory bowel disease Gene Ther 2003 10 854 860 10.1038/sj.gt.3302013 12732871

[B6] Schmid RM Weidenbach H Draenert GF Lerch MM Liptay S Schorr J Liposome mediated in vivo gene transfer into different tissues of the gastrointestinal tract Z Gastroenterol 1994 32 665 670 7871855

[B7] Lau C Soriano HE Ledley FD Finegold MJ Wolfe JH Birkenmeier EH Retroviral gene transfer into the intestinal epithelium Hum Gene Ther 1995 6 1145 1151 10.1089/hum.1995.6.9-1145 8527472

[B8] Noel RA Shukla P Henning SJ Optimization of gene transfer into intestinal epithelial cells using a retroviral vector J Pediatr Gastroenterol Nutr 1994 19 43 49 7965476 10.1097/00005176-199407000-00007

[B9] Lozier JN Yankaskas JR Ramsey WJ Chen L Berschneider H Morgan RA Gut epithelial cells as targets for gene therapy of hemophilia Hum Gene Ther 1997 8 1481 1490 10.1089/hum.1997.8.12-1481 9287148

[B10] Laine F Blouin V Ferry N Evaluation of recombinant retrovirus and adenovirus for gene transfer to normal and pathologic intestinal tissue Gastroenterol Clin Biol 1999 23 221 228 10353017

[B11] Kesisoglou F Schmiedlin-Ren P Fleisher D Roessler B Zimmermann EM Restituting intestinal epithelial cells exhibit increased transducibility by adenoviral vectors J Gene Med 2006 8 1379 1392 10.1002/jgm.981 17133338

[B12] Brown GR Thiele DL Silva M Beutler B Adenoviral vectors given intravenously to immunocompromised mice yield stable transduction of the colonic epithelium Gastroenterology 1997 112 1586 1594 10.1016/S0016-5085(97)70040-4 9136837

[B13] Russell WC Update on adenovirus and its vectors J Gen Virol 2000 81 2573 2604 11038369 10.1099/0022-1317-81-11-2573

[B14] Bessis N GarciaCozar FJ Boissier MC Immune responses to gene therapy vectors: influence on vector function and effector mechanisms Gene Ther 2004 11 Suppl 1 S10 S17 10.1038/sj.gt.3302364 15454952

[B15] Polyak S Mah C Porvasnik S Herlihy JD Campbell-Thompson M Byrne BJ Gene delivery to intestinal epithelial cells in vitro and in vivo with recombinant adeno-associated virus types 1, 2 and 5 Dig Dis Sci 2008 53 1261 1270 10.1007/s10620-007-9991-1 17934813 PMC3896329

[B16] Bukrinsky MI Haffar OK HIV-1 nuclear import: in search of a leader Front Biosci 1999 4 D772 D781 10.2741/Bukrinsky 10525473

[B17] Zennou V Petit C Guetard D Nerhbass U Montagnier L Charneau P HIV-1 genome nuclear import is mediated by a central DNA flap Cell 2000 101 173 185 10.1016/S0092-8674(00)80828-4 10786833

[B18] Schubert M Joshi B Blondel D Harmison GG Insertion of the human immunodeficiency virus CD4 receptor into the envelope of vesicular stomatitis virus particles J Virol 1992 66 1579 1589 1310767 10.1128/jvi.66.3.1579-1589.1992 PMC240885

[B19] Blomer U Naldini L Verma IM Trono D Gage FH Applications of gene therapy to the CNS Hum Mol Genet 1996 5 Spec No 1397 1404 8875243 10.1093/hmg/5.supplement_1.1397

[B20] Sakoda T Kasahara N Hamamori Y Kedes L A high-titer lentiviral production system mediates efficient transduction of differentiated cells including beating cardiac myocytes J Mol Cell Cardiol 1999 31 2037 2047 10.1006/jmcc.1999.1035 10591030

[B21] Shichinohe T Bochner BH Mizutani K Nishida M Hegerich-Gilliam S Naldini L Development of lentiviral vectors for antiangiogenic gene delivery Cancer Gene Ther 2001 8 879 889 10.1038/sj.cgt.7700388 11773978

[B22] Borok Z Harboe-Schmidt JE Brody SL You Y Zhou B Li X Vesicular stomatitis virus G-pseudotyped lentivirus vectors mediate efficient apical transduction of polarized quiescent primary alveolar epithelial cells J Virol 2001 75 11747 11754 10.1128/JVI.75.23.11747-11754.2001 11689655 PMC114760

[B23] Li W Nadelman C Gratch NS Li W Chen M Kasahara N An important role for protein kinase C-delta in human keratinocyte migration on dermal collagen Exp Cell Res 2002 273 219 228 10.1006/excr.2001.5422 11822877

[B24] Chen M Li W Fan J Kasahara N Woodley D An efficient gene transduction system for studying gene function in primary human dermal fibroblasts and epidermal keratinocytes Clin Exp Dermatol 2003 28 193 199 10.1046/j.1365-2230.2003.01191.x 12653712

[B25] Naldini L Blomer U Gage FH Trono D Verma IM Efficient transfer, integration, and sustained long-term expression of the transgene in adult rat brains injected with a lentiviral vector Proc Natl Acad Sci USA 1996 93 11382 11388 10.1073/pnas.93.21.11382 8876144 PMC38066

[B26] Kafri T Blomer U Peterson DA Gage FH Verma IM Sustained expression of genes delivered directly into liver and muscle by lentiviral vectors Nat Genet 1997 17 314 317 10.1038/ng1197-314 9354796

[B27] Miyoshi H Smith KA Mosier DE Verma IM Torbett BE Transduction of human CD34+ cells that mediate long-term engraftment of NOD/SCID mice by HIV vectors Science 1999 283 682 686 10.1126/science.283.5402.682 9924027

[B28] Johnson LG Olsen JC Naldini L Boucher RC Pseudotyped human lentiviral vector-mediated gene transfer to airway epithelia in vivo Gene Ther 2000 7 568 574 10.1038/sj.gt.3301138 10819571

[B29] Kremer KL Dunning KR Parsons DW Anson DS Gene delivery to airway epithelial cells in vivo: a direct comparison of apical and basolateral transduction strategies using pseudotyped lentivirus vectors J Gene Med 2007 9 362 368 10.1002/jgm.1025 17380490

[B30] Seppen J Barry SC Klinkspoor JH Katen LJ Lee SP Garcia JV Apical gene transfer into quiescent human and canine polarized intestinal epithelial cells by lentivirus vectors J Virol 2000 74 7642 7645 10.1128/JVI.74.16.7642-7645.2000 10906219 PMC112286

[B31] Fletcher PS Elliott J Grivel JC Margolis L Anton P McGowan I Ex vivo culture of human colorectal tissue for the evaluation of candidate microbicides AIDS 2006 20 1237 1245 10.1097/01.aids.0000232230.96134.80 16816551

[B32] Koya RC Kimura T Ribas A Rozengurt N Lawson GW Faure-Kumar E Lentiviral vector-mediated autonomous differentiation of mouse bone marrow cells into immunologically potent dendritic cell vaccines Mol Ther 2007 15 971 980 10.1038/mt.sj.6300126 17375074

[B33] Zufferey R Dull T Mandel RJ Bukovsky A Quiroz D Naldini L Self-inactivating lentivirus vector for safe and efficient in vivo gene delivery J Virol 1998 72 9873 9880 9811723 10.1128/jvi.72.12.9873-9880.1998 PMC110499

[B34] Dull T Zufferey R Kelly M Mandel RJ Nguyen M Trono D A third-generation lentivirus vector with a conditional packaging system J Virol 1998 72 8463 8471 9765382 10.1128/jvi.72.11.8463-8471.1998 PMC110254

[B35] Landazuri N Le Doux JM Complexation of retroviruses with charged polymers enhances gene transfer by increasing the rate that viruses are delivered to cells J Gene Med 2004 6 1304 1319 10.1002/jgm.618 15495270

[B36] Vallance BA Gunawan MI Hewlett B Bercik P Van KC Galeazzi F TGF-beta1 gene transfer to the mouse colon leads to intestinal fibrosis Am J Physiol Gastrointest Liver Physiol 2005 289 G116 G128 10.1152/ajpgi.00051.2005 15778431

[B37] Wallace JL Keenan CM Gale D Shoupe TS Exacerbation of experimental colitis by nonsteroidal anti-inflammatory drugs is not related to elevated leukotriene B4 synthesis Gastroenterology 1992 102 18 27 1309357 10.1016/0016-5085(92)91779-4

[B38] Cooper HS Murthy SN Shah RS Sedergran DJ Clinicopathologic study of dextran sulfate sodium experimental murine colitis Lab Invest 1993 69 238 249 8350599

[B39] Sastry L Johnson T Hobson MJ Smucker B Cornetta K Titering lentiviral vectors: comparison of DNA, RNA and marker expression methods Gene Ther 2002 9 1155 1162 10.1038/sj.gt.3301731 12170379

[B40] Stripecke R Koya RC Ta HQ Kasahara N Levine AM The use of lentiviral vectors in gene therapy of leukemia: combinatorial gene delivery of immunomodulators into leukemia cells by state-of-the-art vectors Blood Cells Mol Dis 2003 31 28 37 10.1016/S1079-9796(03)00062-7 12850480

[B41] Seppen J Barry SC Klinkspoor JH Katen LJ Lee SP Garcia JV Apical gene transfer into quiescent human and canine polarized intestinal epithelial cells by lentivirus vectors J Virol 2000 74 7642 7645 10.1128/JVI.74.16.7642-7645.2000 10906219 PMC112286

[B42] Schmiedlin-Ren P Kesisoglou F Mapili JA Sabek SE Barnett JL Chey WD Increased transduction of human intestinal epithelial cells by adenoviral vectors in inflammatory bowel disease Inflamm Bowel Dis 2005 11 464 472 10.1097/01.MIB.0000158535.54428.a5 15867586

[B43] Yee JK Friedmann T Burns JC Generation of high-titer pseudotyped retroviral vectors with very broad host range Methods Cell Biol 1994 43 Pt A 99 112 full_text 7823872 10.1016/s0091-679x(08)60600-7

[B44] Sharma S Cantwell M Kipps TJ Friedmann T Efficient infection of a human T-cell line and of human primary peripheral blood leukocytes with a pseudotyped retrovirus vector Proc Natl Acad Sci USA 1996 93 11842 11847 10.1073/pnas.93.21.11842 8876225 PMC38146

