# Supplementary figures and images for: Effective in vivo and ex vivo gene transfer to intestinal mucosa by VSV-G-pseudotyped lentiviral vectors
Source: BMC Gastroenterol. 2010 May 11;10:44. doi: 10.1186/1471-230X-10-44 (PMC2881878; doi:10.1186/1471-230X-10-44)

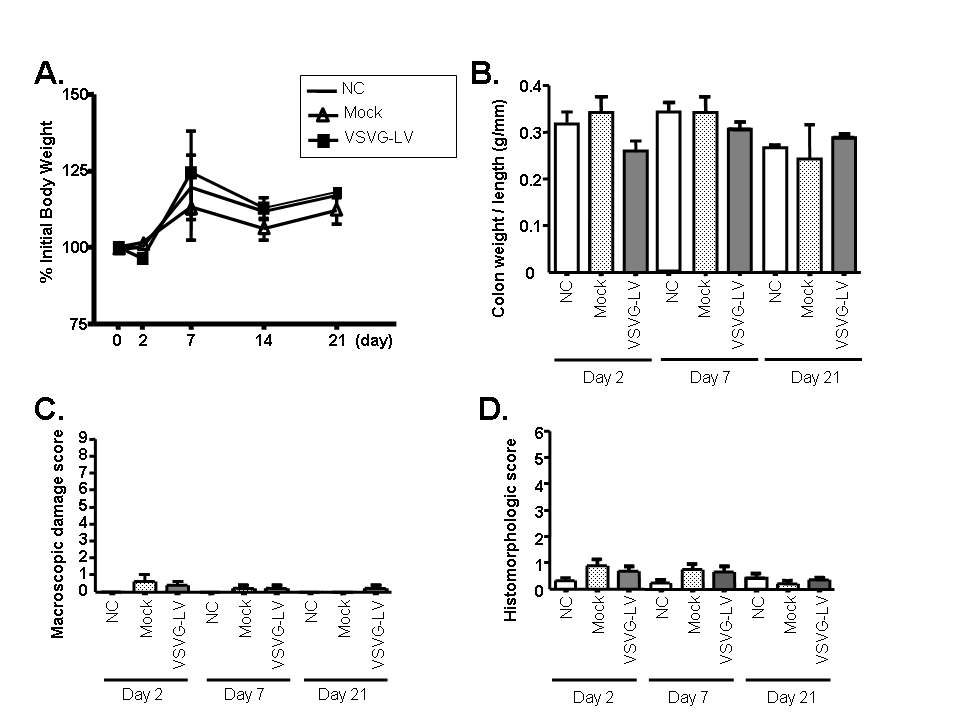

Supplement: Additional file 1 — The effect of vesicular stomatitis virus G protein (VSV-G)-pseudotyped lentivirus (LV) rectal gene transduction on healthy murine cells. BALB/c mice were divided into three groups; a normal healthy control (NC) group and two groups that received either placebo or VSV-G LV following a preliminary ethanol enema (EtOH). Mice received 1000 ng p24 VSV-G LV by rectal administration under anesthesia. (A) VSV-G LV did not affect weight loss in healthy mice. Results are shown as a percentage of original weight for each group. (B) There was no significant difference in the ratio of colon length to weight between groups. Further, there was no significant difference in (C) macroscopic damage score or (D) histomorphologic score among all groups. [file 1471-230X-10-44-S1.TIFF]

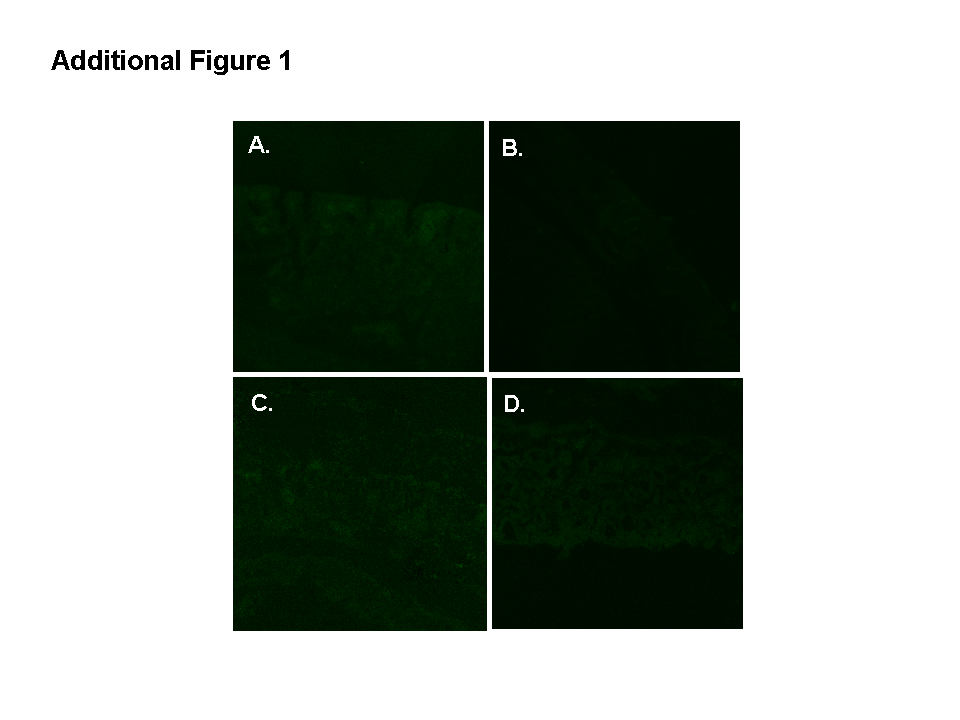

Supplement: Additional file 2 — The negative control pictures of immunofluorence staining using AF488 secondary antibody. Non-specific green staining is not observed in either (A) AE1/AE3 or (B) CD45 examination of murine intestinal tissue or (C) AE1/AE3 (D) CD45 examination of ex vivo human explant tissue [file 1471-230X-10-44-S2.TIFF]
